# A new *GNPAT* variant of foetal rhizomelic chondrodysplasia punctata

**DOI:** 10.1002/mgg3.1733

**Published:** 2021-06-10

**Authors:** Adalgisa Cordisco, Elisabetta Pelo, Mariarosaria Di Tommaso, Roberto Biagiotti

**Affiliations:** ^1^ Division of Prenatal Diagnosis Center P. Palagi Hospital Florence Italy; ^2^ Department of Genetic Diagnosis Careggi Hospital Florence Italy; ^3^ Division of Obstetrics and Gynecology Department of Health Sciences University of Florence Florence Italy; ^4^ Division of Prenatal Diagnosis Meyer Children's Hospital Florence Italy

**Keywords:** *GNPAT*, prenatal diagnosis, rhizomelic chondrodysplasia punctata

## Abstract

**Background:**

Rhizomelic chondrodysplasia punctata (RCDP) is a clinical entity resulting from defects of peroxisomal metabolism whose clinical phenotype is characterized by rhizomelia, calcified foci in periarticular cartilage, coronal lesions of vertebral bodies, cataracts and severe cognitive delay. Usually, survival does not exceed the first decade of life. Transmission is autosomal recessive and is related to mutations in the *PEX7*, *GNPAT* or *AGPS*.

**Methods:**

A detailed description of the prenatal ultrasound signs of RCDP found in two successive pregnancies in a consanguineous couple is reported. Molecular genetic investigations included the study of the coding regions and the exon–intron junctions of the *GNPAT* (high‐throughput amplification and sequencing performed with Roche NimbleGen SeqCap Target kit on Illumina platform); the confirmation test was carried out by amplification and Sanger sequencing with automatic capillary sequencer.

**Results:**

In addition to the typical prenatal ultrasound signs described in the literature in association with RCDP, the presence of prefrontal oedema, never previously described, has been detected in both pregnancies. Moreover, genetic investigations have found a new splicing variant c.924+1G>A of the homozygous *GNPAT*.

**Conclusion:**

The role of mutation in the *GNPAT* suggests a likely association with the clinical phenotype.

## Graphical abstract

Rhizomelic chondrodysplasia punctata (RCDP) is a rare developmental disorder, characterized by rhizomelic bone shortening and epiphyseal stippling, microcephaly, characteristic facial features and severe psychomotor retardation. Transmission is autosomal recessive and is related to mutations in the *PEX7*, *GNPAT* or *AGPS* genes. A fetal diagnosis of RCDP with a new mutation of the *GNPAT* gene is reported.

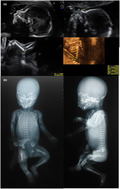

Rhizomelic chondrodysplasia punctata (RCDP) is a clinical entity resulting from defects of peroxisomal metabolism whose clinical phenotype is characterized by rhizomelia, calcified foci in periarticular cartilage, coronal lesions of vertebral bodies, cataracts, severe cognitive delay. Usually, survival does not exceed the first decade of life. Transmission is autosomal recessive and is related to mutations in the *PEX7* (OMIM *601757 *PEX7*), *GNPAT* (OMIM *602744 *GNPAT*) or *AGPS* (OMIM *603051 *AGPS*). A new mutation of the *GNPAT* causing rhizomelic chondrodysplasia is reported.

We followed the two consecutive pregnancies of Mrs C.D. with foetuses affected by RCDP at our Prenatal Diagnosis Center.

During the first pregnancy, the patient was referred to our Prenatal Diagnosis Center at 21 weeks, after recording shortness of both humeri during the foetal morphological scan (25.6 mm corresponding to <1st percentile; Z score −3.831 (Chitty & Altman, 2002 Aug)). At the second level ultrasound examination at 21 + 4 weeks, the humerus length was in line with the measure previously detected, considering the normal range of variability inherent in the methodology (24.5 mm corresponding to <1st percentile; Z score −4.917 (Chitty & Altman, 2002) and, in addition, calcifications at the humeral epiphysis and prefrontal oedema on a mid‐sagittal view of foetal profile (7 mm, corresponding to >99th percentile (Levaillant et al., 2005) were detected. Prefrontal oedema was found. No further abnormalities emerged; in particular, the length of radius, ulna, femur, tibia and fibula remained within the reference percentiles for the gestational age. The rhizomelia associated with the presence of epiphyseal calcifications led the suspect to a severe form of RCDP. After genetic counselling the couple decided to terminate the pregnancy. A late amniocentesis was performed in order to initiate targeted molecular genetic investigations. Foetal autopsy confirmed the shortness of the humeri with regular representation of the other skeletal segments, regular skull and chest conformation. The microscopic examination did not reveal any other associated organ abnormalities.

Despite the contrary opinion of health care professionals, the couple embarked on a new pregnancy before waiting for the outcome of the molecular genetic investigations on the amniotic fluid of the first foetus. However the second pregnancy was followed early by our Prenatal Diagnosis Center; during the first‐trimester scan a nuchal translucency >95th percentile was detected (3.10 mm at 12 + 2 weeks) with a positive screening test for Down syndrome (1/76); the early foetal morphological scan at 17 + 5 weeks revealed a humerus length towards the lower limits for gestational age (20.5 mm corresponding to 1st percentile; Z score: −2.379 (Chitty & Altman, 2002) with the presence of epiphyseal calcifications; the study of the foetal profile on a mid‐sagittal view also showed prefrontal oedema (4.6 mm, corresponding to >99th percentile (Levaillant et al., 2005). These elements made the picture perfectly superimposable on that of the previous pregnancy, allowing us to predict the same evolution. At the ultrasound follow‐up at 19 + 5 weeks the diagnosis of RCDP was clear: the growth of both humeri was significantly slowed down (23.4 mm corresponding to <1st percentile; Z score −3.411 (Chitty & Altman, 2002), epiphyseal calcifications had increased and prefrontal oedema was accentuated (5.5 mm, corresponding to >99th percentile (Levaillant et al., 2005) (Figure 1a). After the new genetic counselling, the couple decided to terminate the pregnancy for the second time, two years after the first interruption. The radiographs of the foetal skeleton (Figure 1b) showed characteristic calcifications on the periarticular cartilage. Foetal autopsy confirmed the shortness of both humeri without other skeletal and organ abnormalities. Due to the strong suspicion of an RCDP, the foetal tissues were analysed for the *PEX7*, *GNPAT* and *AGPS*, known to be involved in RCDP and a splicing variant c.924+1G>A of the homozygous *GNPAT* was found (reference sequence number of *GNPAT*: NM_14236.3).

**FIGURE 1:**
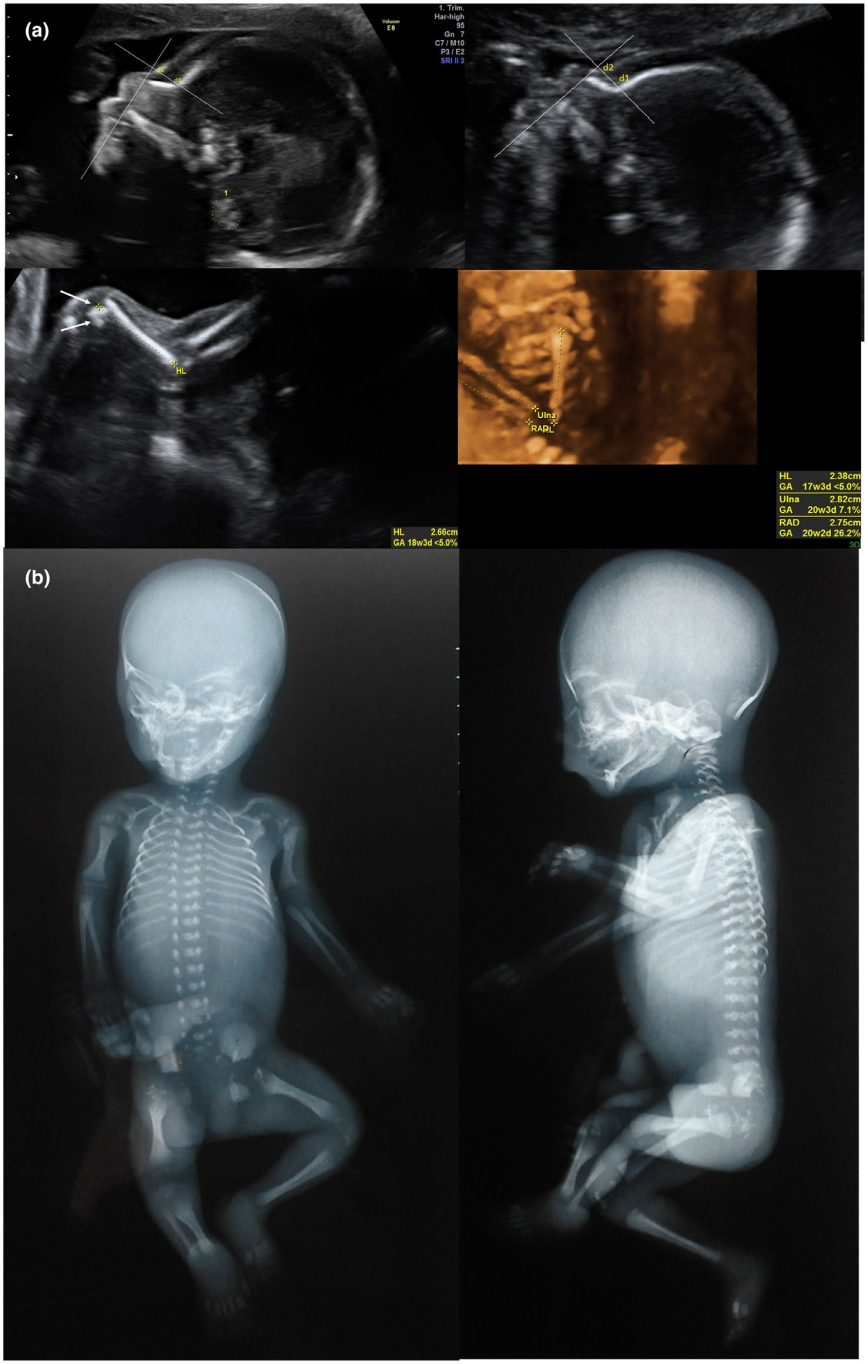
Sagittal ultrasound image of a foetus at 21 weeks 4 days of gestational age (a) and 17 weeks 5 days (a.1) showing prefrontal oedema (d1‐d2) with normal nasal bone and normal frontonasal angle, an important ultrasound sign for the differential diagnosis of the RCDP sub‐type. Longitudinal 2D (a.2) and 3D (a.3) ultrasound image of the upper long bones at 21 weeks 4 days of gestational age showing the rhizomelia and the epiphyseal stippling (arrows). Radio and ulna biometry is in accordance with the gestational age. (a.2;a.3), (b;b.1) Postnatal radiographs confirming the shortness of both humeri and the presence of calcified punctures in humeral periarticular cartilage. RCDP, Rhizomelic chondrodysplasia punctata

Chondrodysplasia punctata is a rare, multisystem, developmental disorder first described by Erich Conradi. Type 1 RCDP (OMIM *215100) is caused by homozygous or compound heterozygous mutation in the *PEX7*, which encodes the peroxisomal type 2 targeting signal receptor on chromosome 6q23. Type 2 RCDP (OMIM *222765) is caused by a mutation in the gene encoding acyl‐CoA:dihydroxyacetonephosphate acyltransferase (*GNPAT*) on chromosome 1q42. Type 3 RCDP (OMIM *600121) is caused by mutation in the gene encoding alkyldihydroxyacetonephosphate synthase (alkyl‐DHAP synthase) on chromosome 2q31. Type 5 RCDP (OMIM *616716) is caused by mutation in the gene encoding peroxisomal biogenesis factor‐5 (OMIM *600414 *PEX5*) on chromosome 12p13 (OMIM® Online Mendelian Inheritance in Man®; Waterham & Ebberink, 2012). While RCDP1 is a peroxisomal biogenesis disorder, RCDP2 and RCDP3 are classified as single peroxisome enzyme deficiencies (Heymans et al., 1985; Waterham & Ebberink, 2012). Prevalence of the rhizomelic type is estimated at 1 in 100,000 live births. Postnatal diagnosis is based on clinical and radiological findings and can be confirmed by molecular analysis. Prenatal diagnosis is possible when the causative mutation has already been identified in the family. RCDP has a severe prognosis with death generally occurring during the first decade of life, mainly due to respiratory complications. Clinically speaking, RCDP is characterized by severe bilateral shortening and metaphyseal changes of femora and/or humeri, microcephaly, characteristic facial features including a broad nasal bridge, epicanthus, high‐arched palate, dysplastic external ears and micrognathia, congenital contractures, characteristic ocular involvement and severe psychomotor retardation and spasticity. There are several different disorders with similar punctate cartilaginous changes, for example X‐linked chondrodysplasia punctate, the multiple forms of the Zellweger syndrome, maternal ingestion of certain anticoagulants (dicoumarol or warfarin) in early pregnancy and even occasionally trisomy 18.

There are only few reports of prenatal diagnosis of RCDP in the Medline literature (Başbuğ et al., 2005; Brookhyser et al., 1999; Duff et al., 1990 Sep; Erdogdu et al., 2016; Gendall et al., 1994; Hertzberg et al., 1999; Sastrowijoto et al., 1994). The pattern recognition is based on the combination of rhizomelic bone shortening and epiphyseal stippling. An author suggested the role of bilateral cataracts in suspecting the diagnosis (Gendall et al., 1994). A recent literature review highlights how the prenatal diagnosis of epiphyseal stippling as well as spinal and extremity abnormalities (hands and feet) is often missed (Blask et al., 2018). To date, no cases of prenatal diagnosis of rhizomelic forms of chondrodysplasia related to *GNPAT* variants have been reported.

Although the diagnosis of this type of chondrodysplasia is genetic, we believe that it is important already in the prenatal period to try to define the subtype of suspected chondrodysplasia because the associated prognosis varies considerably: from mild forms with a good life expectancy to very severe forms. Based on the diagnostic signs reported in the literature we can affirm that the main unfavourable prognostic factor is the presence of rhizomelia (typical of type 2, 3 and 5 RCDP) while the most favourable prognostic factors are the symmetrical shortness of long bones and the nose/maxillary hypoplasia known as Binder phenotype. Table 1 summarizes the published works on prenatal diagnosis of RCDP with the related ultrasound signs.

**TABLE 1:** The published works on prenatal diagnosis of RCDP with the related ultrasound signs

Year of publication	Authors	Gestational age (weeks)	Ultrasound signs
1990	Duff P, Harlass FE, Milligan DA	28	Stippling at the proximal end of the right humerus as well as in the proximal portion of both femurs
1994	Sastrowijoto SH, Vandenberghe K, Moerman P, Lauweryns JM, Fryns JP	19	Bilateral symmetric proximal shortening of the humeri and of the femurs below the third percentile. Marked epiphyseal echo‐reflectivity, brachycephaly, hydrocephalus with tetraventricular dilatation, scal oedema and hypertelorism
1994	Gendall PW, Baird CE, Becroft DM	25	Twin pregnancy Twin 1: rhizomelic limb shortening, stippling at the proximal end of both humeri as well as in the proximal portion of both femurs
1999	Hertzberg BS, Kliewer MA, Decker M, Miller CR, Bowie JD	28	Bilateral symmetric shortening of the humeri and of the femurs, stippling at the proximal and distal epiphyses o the long bones, marked flaring of the distal methaphysis of both humeri
2005	Başbuğ M, Serin IS, Ozçelik B, Guneş T, Akçakuş M, Tayyar M	30 32 34	Rhizomelic limb shortening and polydramnios Bilateral cataracts Stippling at proximal epiphyses of femur and humerus
2010	Zwijnenburg PJ, Deurloo KL, Waterham HR, Meijers‐Heijboer EJ, van Vugt JM, Tan‐Sindhunata MB	19 + 6 21 + 6	Shortening of both humeri (below 5th percentile) with stippling in the epiphyseal catilage Bowing of both humeri, shortening of both femurs with stippling in the epiphyseal catilage

Abbreviation: RCDP, Rhizomelic chondrodysplasia punctate.

In the reported case, the circumstance of having followed two affected foetuses in two successive pregnancies allowed the obtainment of perfectly overlapping images with highly specific signs of RCDP: shortness of both humeri, prefrontal oedema, epiphyseal stippling (Figure 1a). There was no saddling of the root of the nose with Binder phenotype, no spine irregularity, no brachytelephalangy and no cataracts. The length of the other skeletal segments was in accordance with the gestational age. Counselling was therefore directed towards a form of dysplasia with a more severe prognosis (unlike the more frequent type 1).

For the first time, the presence of prefrontal oedema (defined as the distance from nasal bone synostosis towards the front following an orthogonal angle at 45° with calipers set on to on (Levaillant et al., 2005) has been described as an ultrasound sign associated with RCDP (Figure 1a). This space was 7 mm at 21 weeks (corresponding to >95th percentile [Levaillant et al., 2005 Dec]) during the scan examination in the first pregnancy and 4.6 mm at 17 weeks (corresponding to >95th percentile (Levaillant et al., 2005) during the scan performed in the second pregnancy (Figure 1a). The thickness of the prefrontal space has been described in the literature as an ultrasound marker of Down's syndrome (De Jong‐Pleij et al., 2012; Persico et al., 2008). However, it represents a non‐specific ultrasound sign, which is also common to different genetic diseases. In the proposed case, its presence in two foetuses affected by the same type of RCDP suggests that the modification of prefrontal connective tissue could be a phenotypic sign associated with the new genetic variant described in the *GNPAT*. However, the small number of cases requires us to be cautious and to wait for further reports.

Genetic investigations included the study of the coding regions and the exon–intron junctions of the *GNPAT* (high‐throughput amplification and sequencing performed with Roche NimbleGen SeqCap Target kit on Illumina platform); the confirmation test was carried out by amplification and Sanger sequencing with automatic capillary sequencer. A new splicing variant c.924+1G>A of the homozygous *GNPAT* was found (cDNA Level: NM_14236.3:c.924+1G>A; gDNA Level: Chr1(GRCh37):g.231401912G>A). Both parents presented this heterozygous variant.

The identified variant has not been identified in the literature and is not annotated on public databases of variant reporting (ClinVar, LOVD, etc.). Nevertheless, according to ACMG 2015 recommendations for variant interpretation (Richards et al., 2015), at present, it can be classifiable as pathogenic applying the following criteria:

- Very strong evidence of pathogenicity (PVS1): null variant (intronic within ±2 of splice site) in *GNPAT* for which loss‐of‐function is a known mechanism of disease (gene has 7 pathogenic Loss‐of‐Function (LOF) variants and gnomAD LOF Observed/Expected = 0.231 is less than 0.763), associated with type 2 RCDP.
- Moderate evidence of pathogenicity (PM2): variant not found in gnomAD exomes (good gnomAD exomes coverage = 95.3). Variant not found in gnomAD genomes (good gnomAD genomes coverage = 32.9).
- Supporting evidence of pathogenicity (PP3): pathogenic computational verdict based on six pathogenic predictions from BayesDel_addAF, DANN, EIGEN, FATHMM‐MKL, MutationTaster and scSNV‐Splicing vs no benign predictions.

In conclusion, the role of the homozygous splicing variant c.924+1G>A in the *GNPAT*, not reported in the scientific literature, suggests a likely association with the clinical phenotype.

## CONFLICT OF INTEREST

The authors have no conflicts of interest to declare.

## STATEMENT OF ETHICS

The research was conducted ethically in accordance with the World Medical Association Declaration of Helsinki. The patient has given her written informed consent to publish her case (including publication of images).

## AUTHORS’ CONTRIBUTIONS

Adalgisa Cordisco contributed in writing and reviewing the paper; Elisabetta Pelo contributed in reviewing paper; Mariarosaria Di Tommaso contributed in reviewing paper; Roberto Biagiotti contributed in reviewing paper.

## Data Availability

Data sharing not applicable to this article as no datasets were generated or analysed during the current study.
